# Nalbuphine suppresses breast cancer stem-like properties and epithelial-mesenchymal transition via the AKT-NFκB signaling pathway

**DOI:** 10.1186/s13046-019-1184-1

**Published:** 2019-05-15

**Authors:** Jiachuan Yu, Yuanyuan Luo, Qingping Wen

**Affiliations:** 1 Department of Anesthesiology, The First Affiliated Hospital of Dalian Medical University, Zhongshan Road, Dalian, 116011 China; 2 Institute of Cancer Stem Cell, Dalian Medical University, Dalian, 116044 China

**Keywords:** Nalbuphine, Breast cancer, Stem-like traits, Epithelial-mesenchymal transition, AKT-NFκB pathway

## Abstract

**Background:**

Cancer pain is a debilitating disorder of human breast cancer and a primary determinant of the poor quality of life, and relieving pain is fundamental strategy in the cancer treatment. However, opioid analgesics, like morphine and fentanyl, which are widely used in cancer pain treatment have been reported to enhance stem-like traits and epithelial-mesenchymal transition (EMT) of breast cancer cells. As such, it is vital to make the best choice of analgesic for breast cancer management.

**Methods:**

MTT assays and colony formation assays were performed to examine tumor cell proliferation upon nalbuphine treatment. RT-PCR, western blot, flow cytometry, sphere formation, immunohistochemistry, transwell assays, wound healing assays and mouse xenograft were used to assess the biological effects of nalbuphine treatment.

**Results:**

Nalbuphine inhibited breast cancer cell growth and tumorigenesis, with little effect on noncancerous breast cell lines. Nalbuphine suppressed cancer stem-like traits and EMT in both breast cancer cells and mouse xenograft tumor tissues. Additionally, activation of AKT reversed the nalbuphine-induced inhibition of cancer stem-like properties, tumorigenesis and EMT.

**Conclusions:**

Our results demonstrate a new role of nalbuphine in inhibiting cancer stem-like properties and EMT in addition to relieving pain, which suggests that nalbuphine may be effective in breast cancer treatment.

**Electronic supplementary material:**

The online version of this article (10.1186/s13046-019-1184-1) contains supplementary material, which is available to authorized users.

## Background

A large number of breast cancer patients suffer from cancer-related pains caused by surgery, tumor progression and bone metastasis [1]. This leads to the patients’ constant demand for analgesic treatment during breast cancer therapy. Our previous work showed that morphine and fentanyl, the classic opioid analgesics, promoted breast cancer stem-like traits and epithelial-mesenchymal transition (EMT) [2, 3]. Some previous studies [4–6] also proved that opioid analgesics could induce cancer metastasis, angiogenesis, and drug resistance. However, nalbuphine, a narcotic kappa receptor agonist and partial mu receptor antagonist, provides comparable analgesic efficacy to opioid analgesics [7] with fewer opioid-induced adverse effects [8]. It has been shown to be safe and effective when used for the treatment of conditions ranging from burns, multiple trauma, orthopedic injuries, gynecological and intra-abdominal conditions [9, 10]. Although nalbuphine has been proven to be potent and reliable against acute and chronic pain, its influence on the stem-like traits and EMT of human breast cancer cells has not been elucidated.

Cancer stem cells (CSCs) are part of a cellular hierarchy, harboring stem cell-like properties of self-renewal combined with the potential for aberrant differentiation [11, 12]. The presence of CSCs is closely associated with tumorigenesis [13] and drug resistance [14], and they are able to generate the heterogeneous lineages of cancer cells that comprise tumors. Our previous results demonstrated that morphine and fentanyl could promote the development of breast cancer cells with stem-like phenotypes [2, 3], but little is known about the effect of nalbuphine on cancer stem-like properties. The epithelial-mesenchymal transition (EMT) is a complex cellular program by which epithelial cells lose their epithelial features and are transformed into cells with a mesenchymal phenotype [15]. It is well known that EMT is associated with embryonic development and wound healing, but the phenotype is also inherent to tumor invasiveness and metastasis, as well as stemness [16]. Whether nalbuphine can affect EMT in breast cancer remains largely unknown. Accumulated genetic and cell biology evidence demonstrates that the AKT-NFκB pathway is a central mechanism controlling EMT and the CSC phenotype [17–22], and drugs that inhibit the AKT-NFκB pathway might yield promising therapeutic effects. However, the effects of nalbuphine on the AKT-NFκB signaling pathway, and further influences on stem-like properties and EMT in breast cancer have not been investigated.

In this study, we demonstrated that nalbuphine inhibited stem-like traits and EMT of the MDA-MB-231, MCF-7 and SK-BR-3 cell lines through the suppression of AKT-NFκB signaling. Our findings suggest that nalbuphine may be a better choice for breast cancer pain treatment than traditional opioids.

## Methods

### Cell lines and culture conditions

Human cancer cell lines (MDA-MB-231, MCF-7, SK-BR-3, A549, NCI-H460, NCI-H1299, HepG2, AGS, HCT116, HeLa and TT) and normal human mammary epithelial cells (MCF-10A and MCF-10F) were purchased from the American Type Culture Collection (ATCC) and grown according to their recommendations. These cell lines were authenticated at ATCC before purchase by standard short tandem repeat DNA-typing methodology. Human nasopharyngeal carcinoma cell line (CNE1) and human mesenchymal stem cell (MSC) were purchased from BeNa Culture Collection (BNCC). MDA-MB-231, SK-BR-3 and MSC were maintained in Dulbecco’s Modified Eagle’s Medium (DMEM, Invitrogen Corp) supplemented with 10% fetal bovine serum (Invitrogen Corp). MCF-7 was maintained in Eagle’s Minimum Essential Medium (EMEM, Invitrogen Corp) supplemented with 10% fetal bovine serum and 0.01 mg/ml human recombinant insulin. HepG2 and HeLa were maintained in EMEM supplemented with 10% fetal bovine serum. A549, AGS and TT were grown in Ham’s F-12 K (Kaighn’s) Medium (F-12 K, Invitrogen Corp) with 10% fetal bovine serum. NCI-H1299, NCI-H460 and CNE1 were grown in Roswell Park Memorial Institute 1640 Medium (RPMI 1640, Invitrogen Corp) with 10% fetal bovine serum. HCT116 was grown in McCoy’s 5A (Modified) Medium (Invitrogen Corp) with 10% fetal bovine serum. MCF-10A was maintained in Mammary Epithelium Basal Medium (MEBM, Lonza) added with 10 ng/ml hEGF, 5 μg/ml insulin, 0.5 μg/ml hydrocortisone gentamicin and amphotericin-B. Before use, the medium was completed with bovine pit extract (BPE, Lonza) at a final concentration of 0.4%. MCF-10F was cultured in Dulbecco’s Modified Eagle Medium/Ham’s F-12 (Advanced DMEM/F-12, Invitrogen Corp) with 20 ng/ml epidermal growth factor, 0.01 mg/ml insulin, 500 ng/ml hydrocortisone, 5% horse serum, and 100 μg/ml penicillin/streptomycin mixture. All cells were incubated at 37 °C in a humidified incubator containing 5% CO_2_.

### Drugs and reagents

Nalbuphine Hydrochloride Injection (NMPN H20131027) was from Yichang Humanwell Pharmaceutical Co., Ltd. (China). Morphine Hydrochloride and Fentanyl Hydrochloride were obtained from Northeast Pharmaceutical Group (Shenyang, China). SC79 (MedChemExpress, HY-18749) was dissolved in dimethylsulfoxide (DMSO) to a stock concentration of 10 mM and stored at − 20 °C. IGF-1 (PEPROTECH, 100–11) was dissolved in water to a stock concentration of 100 ng/ml and stored at − 80 °C. The concentration of drugs was chosen based upon successful activation/inhibition in previous publications: morphine (10 μM), fentanyl (0.1 μM), SC79 (10 μM) and IGF-1 (100 ng/ml).

### Mammosphere culture

Sphere formation was performed in ultra-low attachment plates (Corning) with Dulbecco’s Modified Eagle Medium/Nutrient Mixture F-12 (DMEM/F-12, Invitrogen Corp) supplemented with 2% B27, 20 ng/ml bFGF, and 20 ng/ml EGF. MDA-MB-231 and MCF-7 cells were seeded at a density of around 2 cells/μl and cultured at 37 °C with 5% CO_2_. After 14 days, spheres greater than 50 μm diameter were counted at 40x magnification using an Olympus microscope. For the limiting dilution assay, dissociated primary cells were seeded in 96-well plates at densities of 2, 4, 8, 16, 32 and 64 cells per well. After 10 days, the percent of wells not containing spheres for each cell density was calculated and plotted against the cells per well and regression lines were plotted [23].

### ALDEFLUOR assay

The ALDEFLUOR kit (STEMCELL Technologies, #01700), which measures aldehyde dehydrogenase (ALDH), an enzyme marker for cancer stem and progenitor cells, was used for identifying the cell population with a high ALDH activity. Cells were suspended in ALDEFLUOR assay buffer containing ALDH substrate (BAAA, 1 μmol/l per 1 × 10^6^ cells) and incubated for 45 min at 37 °C. As a negative control, for each sample of cells an aliquot was treated with 50 mM diethylaminobenzaldehyde (DEAB), a specific ALDH inhibitor. The ALDH1-positive subpopulation was analyzed by FACS (BD FACSCalibur).

### Mouse xenograft assay

Four- to six-week-old Balb/c mice were used in each experimental group. Nalbuphine was administered at 2 mg/kg/d, equivalent to 0.22 mg/kg/d, the anesthesia induction dosage (s.c.) for humans, morphine at 0.714 mg/kg/d for first 15 days and then 1.43 mg/kg/d (s.c.) [24], fentanyl at 0.02 mg/kg/d (s.c.) [3] and SC79 at 0.04 mg/g/d (i.p.) [25]. The control group of mice received an equal volume of PBS. MDA-MB-231 cells (1 × 10^6^ in 1:1 PBS:Matrigel) were injected subcutaneously into both flanks of mice. For serial dilution assays, 10^2^, 10^3^, 10^4^, and 10^5^ primary cells were subcutaneously injected in each dorsal flank. Tumor sizes were measured in perpendicular dimensions using calipers. Volumes were estimated using the formula (a^2^ × b)/2, where a is the shorter of the two dimensions and b is the longer one. The *p*-value was obtained by comparing the control and treatment groups at each time point. The protocol for experimental animals was approved by the Institutional Animal Care and Use Committee of Dalian Medical University and was in accordance with the national guidelines for the care and maintenance of laboratory animals.

### Statistical analysis

Each in vivo and in vitro experiment was performed in triplicate and repeated at least three times. Data were expressed as mean ± SEM. Statistical analyses were performed with SPSS software (version 16.0) or GraphPad Prism 6.0 (GraphPad Software, Inc.). Differences among variables were assessed by two-tailed Student’s *t*-test and ANOVA test. A *p*-value less than 0.05 was considered statistically significant (*p < 0.05, **p < 0.01, ***p < 0.001).

See other materials and methods in Additional file 1: Supplemental methods.

## Results

### Nalbuphine selectively inhibits tumor cell proliferation

**Fig. 1:**
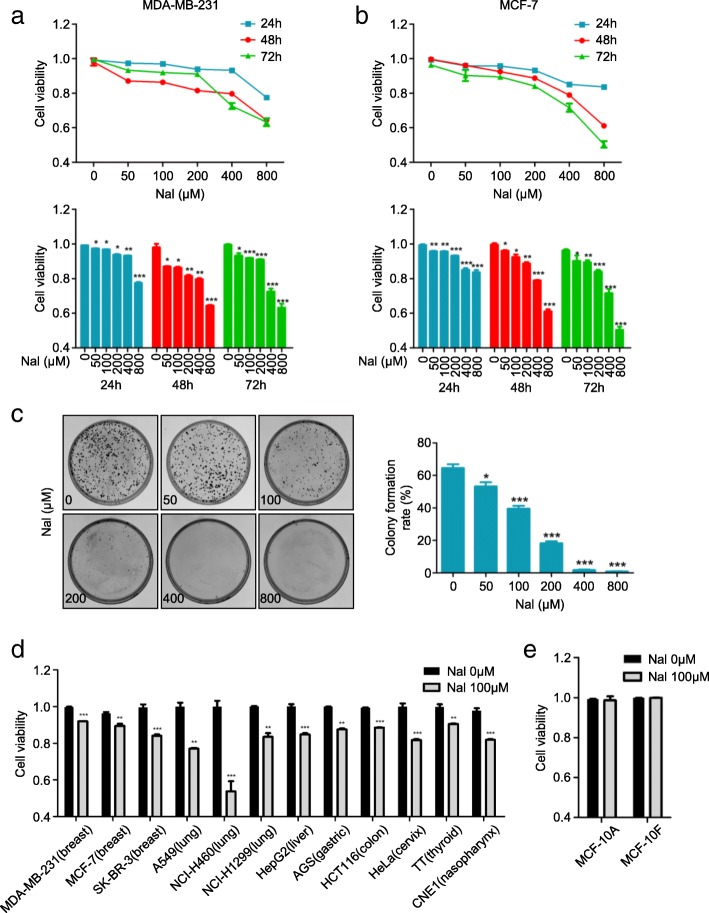
Nalbuphine selectively inhibits tumor cell proliferation. **a, b** MDA-MB-231 and MCF-7 cells were treated with the indicated concentration of nalbuphine (Nal) for the indicated times, and cell viability was measured using the MTT method (*n* = 3). **c** Colony formation of MDA-MB-231 cells treated with indicated concentration of Nal (n = 3). **d** Cell lines derived from various tumor types were treated with 100 μM nalbuphine for 48 h and cell viability was measured by MTT assay (n = 3). **e** MCF-10A and MCF-10F were treated with 100 μM nalbuphine for 48 h and cell viability was measured by MTT assay (n = 3). Data are presented as mean ± SEM. The *p-*value was determined by Student’s *t*-test (*p < 0.05, **p < 0.01, ***p < 0.001). See also Additional file 2: Figure S1

Breast cancer cell lines were incubated with various concentrations of nalbuphine for 24, 48 and 72 h. We found that nalbuphine caused a significant dose- and time-dependent decrease in breast cancer cell viability compared with PBS controls (0 μM) (Fig. 1a, b and Additional file 2: Figure S1A). The 50% inhibitory concentration (IC50) in MDA-MB-231, MCF-7 and SK-BR-3 is listed in Additional file 7: Table S7. These results were further confirmed by colony formation assay. The colony numbers (up to 14 days) were markedly decreased in a dose-dependent manner following nalbuphine treatment (Fig. 1c, Additional file 2: Figure S1B and C). In order to determine whether nalbuphine could induce the death of other tumor cells, cells were incubated with 100 μM nalbuphine for 48 h (based on the findings above and clinical usage,). Nalbuphine treatment led to the death of cell lines derived from various tumor types (Fig. 1d). Importantly, no deleterious effects on the viability of normal human mammary epithelial cells were observed with nalbuphine (Fig. 1e). These findings suggested that nalbuphine selectively inhibits the growth of tumor cells.

### Nalbuphine decreases breast cancer stem-like properties

**Fig. 2:**
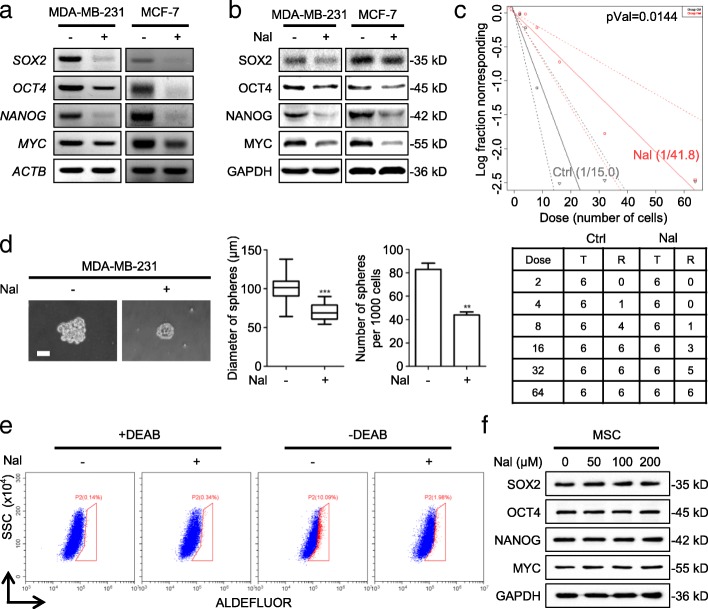
Nalbulphine decreases breast cancer stem-like properties in vitro. **a**, **b** MDA-MB-231 and MCF-7 cells were treated with nalbuphine for 48 h. Levels of indicated mRNA and protein were determined by RT-PCR (**a**) and western blot (**b**) (*n* = 3). **c** MDA-MB-231 cells were treated with nalbuphine for 48 h and then subjected to in vitro limiting dilution analysis. The linear regression plot (*upper*) was generated by ELDA software. Stem cell frequencies are shown in the lower panel (*n* = 3). **d** Representative spheroid images derived from the Ctrl and Nal MDA-MB-231 cells (*upper*) (n = 3); Scale bar, 50 μm. Mammosphere sizes and number of mammospheres (*bottom*) (d > 50 μm) are shown. **e** MDA-MB-231 cells were treated with nalbuphine for 48 h, ALDH activity was then determined with DEAB-treated cells serving as negative controls (*n* = 3). **f** Mesenchymal stem cells were treated with the indicated concentration of nalbuphine for 48 h and levels of the indicated proteins were determined by western blot (n = 3). Data are presented as mean ± SEM. *p*-value was determined by Student’s t-test, Chi-squared test (**c**) and ANOVA (**d**) (*p < 0.05, **p < 0.01, ***p < 0.001). See also Additional file 3: Figure S2

Previous studies indicated that many analgesics modulate stem-like properties of cancer cells [2, 3]. We found that nalbuphine-treated cells expressed significantly lower levels of the self-renewal markers, SOX2, OCT4, NANOG and MYC, as measured by both mRNA (Fig. 2a and Additional file 3: Figure S2A) and protein expression (Fig. 2b and Additional file 3: Figure S2B, C). We also treated cells with nalbuphine on the first day, and then determined the expression of self-renewal markers at 12, 24, 48 and 72 h. The levels of all self-renewal markers decreased at 24 and 48 h, but reverted to the initial level by 72 h (Additional file 3: Figure S2D). In vitro limiting dilution assay showed a reduction in the frequency of sphere-forming cells after treatment of nalbuphine (Fig. 2c). And both diameters and numbers of spheres were decreased after nalbuphine treatment (Fig. 2d and Additional file 3: Figure S2E). The ALDH1-positive sorting assay indicated that the ALDH1^+^ cell population was reduced by nalbuphine treatment (Fig. 2e). Additionally, we examined the effect of nalbuphine on normal stem cell MSC, and the data showed that nalbuphine had no effect on MSC (Fig. 2f).

**Fig. 3:**
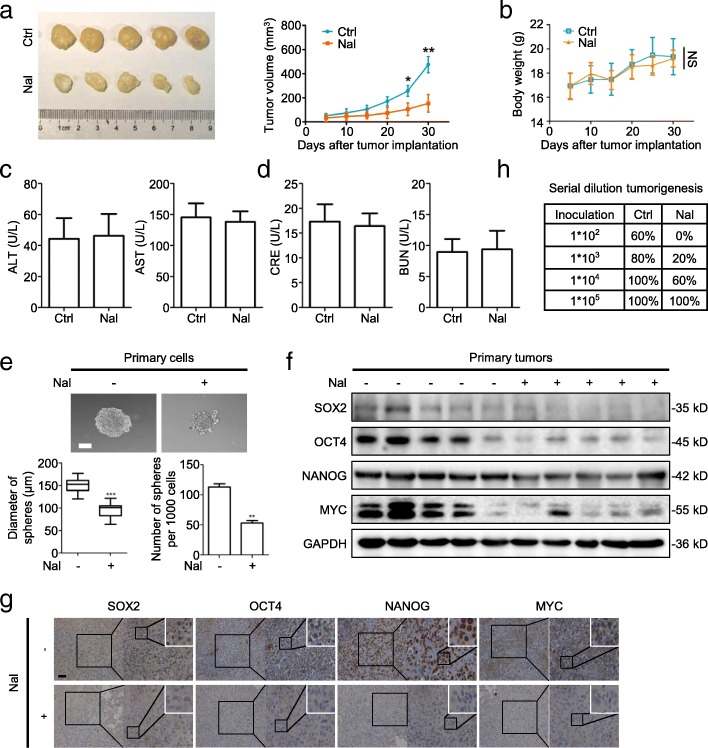
Nalbulphine decreases breast cancer stem-like properties in vivo. **a** Representative tumor image (*left*) from Ctrl and Nal mice. Growth of Ctrl and Nal MDA-MB-231 tumors in mice (*n* = 5). **b** Body weight growth curves of mice over 30 days following tumor implantation (n = 5). **c**, **d** ALT, AST (**c**), CRE and BUN (**d**) levels in serum of Ctrl or Nal mice (n = 5). **e** Representative spheroid images derived from primary Ctrl and Nal tumor cells (*left*) (n = 5); Scale bar, 50 μm. Mammosphere sizes (*upper right*) and numbers of mammospheres (*bottom right*) (d > 50 μm) were shown. **f** Primary cells were extracted from excised Ctrl and Nal tumors. Levels of indicated proteins were determined by western blot (n = 5). **g** Representative IHC staining for indicated proteins in Ctrl and Nal MDA-MB-231 tumor tissue (n = 5); Scale bar, 50 μm. **h** Serial diluted Ctrl MDA-MB-231 tumor cells were subcutaneously inoculated at four different sites in each mouse. Statistical analysis of tumorigenicity with indicated cell numbers and treatments is shown (n = 5). Data are presented as mean ± SEM. *p*-value was determined by Student’s t-test and ANOVA (**a** and **e**) (*p < 0.05, **p < 0.01, ***p < 0.001). See also Additional file 4: Figure S3

Mice were injected daily, s.c., with nalbuphine and xenografted with human breast cancer cells. Nalbuphine suppressed the tumor growth compared to untreated mice (Fig. 3a). The nalbuphine dose used in this study did not decrease the body weight of the mice (Fig. 3b), and no liver or kidney injury was detected (Fig. 3c and d), indicating no significant toxicity. Both the diameter and number of mammospheres derived from the nalbuphine-injected mice were significantly decreased compared with those of PBS-treated control mice (Fig. 3e). Furthermore, nalbuphine-treated tumors expressed significantly lower levels of self-renewal proteins (Fig. 3f). Similar results were also observed by immunohistochemical (IHC) analyses, the nalbuphine-treated group displaying lower expression of stem-like proteins (Fig. 3g). To further confirm that nalbuphine targets the cancer stem-like traits in vivo, serially diluted primary tumor cells were subcutaneously inoculated at 4 different sites into each mouse. Notably, tumor formation rates of nalbuphine-treated mice were reduced. The lowest number of implanted tumor cells (10^2^), nalbuphine decreased tumor formation from 60% in control mice to 0% (Fig. 3h). These data demonstrated that nalbuphine treatment significantly decreased the breast cancer stem-like phenotype. We also determined the effect of other analgesics, morphine and fentanyl, on cancer stem-like traits and tumorigenesis. In accordance with previous studies, both morphine and fentanyl enhanced cancer stemness and tumor growth, while nalbuphine showed the opposite effect (Additional file 4: Figure S3A and B).

### Nalbuphine inhibits EMT and metastasis

**Fig. 4:**
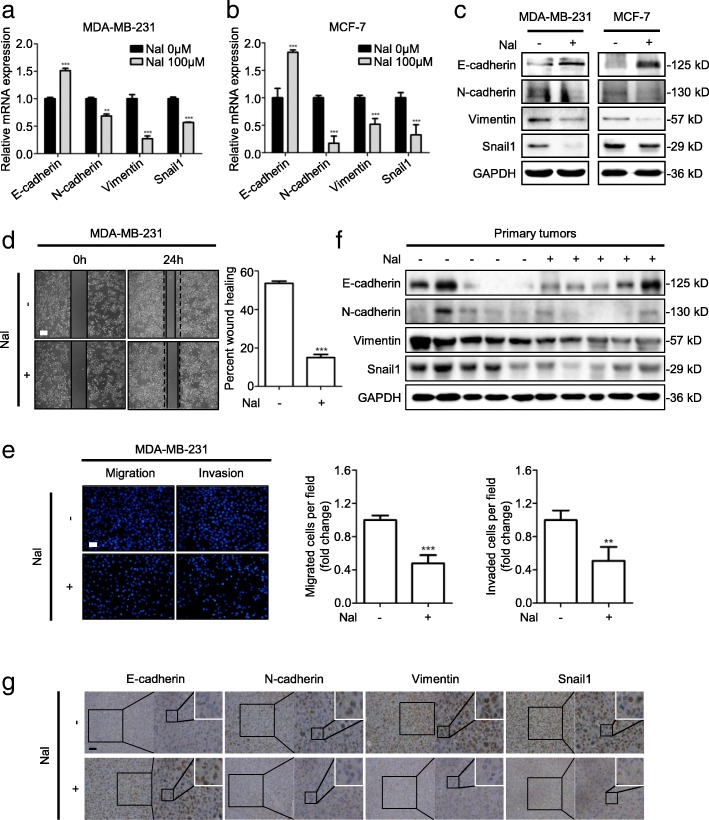
Nalbuphine inhibits EMT and metastasis. **a–c** MDA-MB-231 and MCF-7 cells were treated with nalbuphine for 48 h and levels of indicated mRNA and protein were determined by RT-PCR (**a**, **b**) and western blot (**c**) (n = 3). **d** MDA-MB-231 cells were treated with nalbuphine for 48 h and then subjected to wound healing analysis, representative images (*left*) and statistical analysis (*right*) are shown (n = 3). **e** MDA-MB-231 cells were treated with nalbuphine for 48 h and migration and invasion were then determined by transwell assays. Representative images (*left*) and statistical analysis (*middle and right*) are shown (n = 3). **f** Primary cells were extracted from Ctrl and Nal tumors. Levels of indicated proteins were determined by western blot (n = 5). **g** Primary cells were extracted from Ctrl and Nal tumors. Levels of indicated proteins were determined by western blot (n = 5). Data are presented as mean ± SEM. *p-*value was determined by Student’s *t*-test (*p < 0.05, **p < 0.01, ***p < 0.001). See also Additional file 5: Figure S4

EMT is often associated with cancer stem-like phenotype [26]. Nalbuphine-treated cells showed lower expression of N-cadherin, Vimentin and Snail, but higher expression of E-cadherin in both mRNA (Fig. 4a, b and Additional file 5: Figure S4A) and protein (Fig. 4c and Additional file 5: Figure S4B), compared to control cells. As EMT is a key process in cancer metastasis, we next examined the role of nalbuphine in tumor metastasis by wound healing and transwell assays. Nalbuphine slowed the cell migration from about 55 to 15% (Fig. 4d and Additional file 5: Figure S4C) and significantly blunted cell migration and invasion abilities (Fig. 4e and Additional file 5: Figure S4D). Tumors extracted from nalbuphine-treated mice showed higher level of E-cadherin, and lower levels of N-cadherin, Vimentin and Snail (Fig. 4f) compared with those of control mice. Similar results were observed in IHC analyses (Fig. 4d) verifying that nalbuphine suppressed EMT and tumor metastasis.

### Nalbuphine inhibits breast cancer stem-like properties and EMT through the AKT-NFκB pathway

**Fig. 5:**
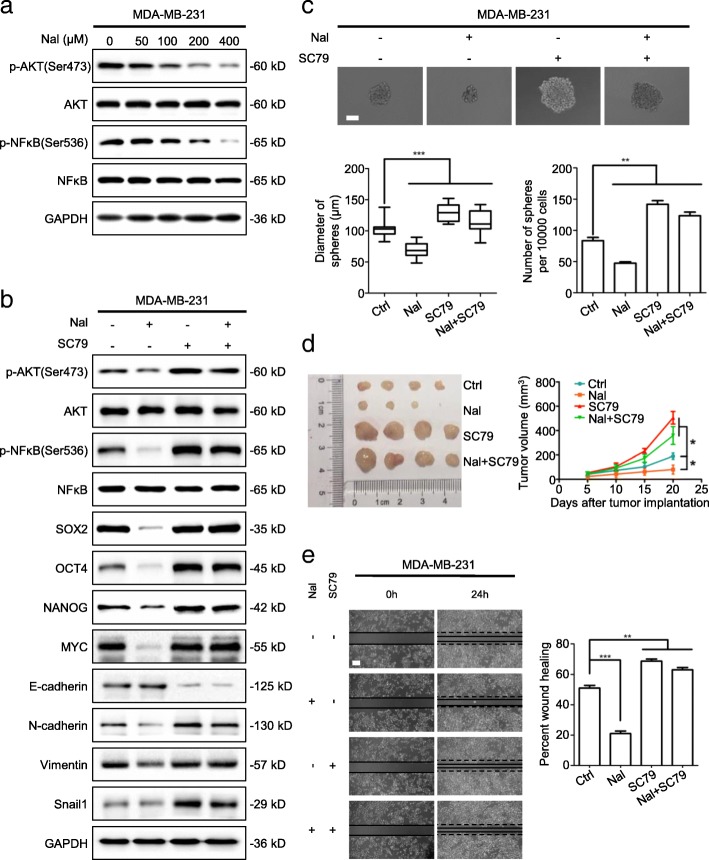
Nalbuphine inhibits breast cancer stem-like properties and EMT through the AKT-NFκB pathway. **a** MDA-MB-231 cells were treated with the indicated concentration of nalbuphine for 48 h and levels of the indicated proteins were determined by western blot (n = 3). **b** MDA-MB-231 cells were treated with nalbuphine and/or SC79 for 48 h and levels of indicated proteins were determined by western blot (n = 3). **c** Representative spheroid images derived from MDA-MB-231 cells treated with nalbuphine and/or SC79 (*upper*) (n = 3); Scale bar, 50 μm. Mammosphere sizes (*bottom left*) and number of mammospheres (*bottom right*) (d > 50 μm) are shown. **d** Representative tumor image (*left)* and growth curves (*right)* of mice with indicated treatment (n = 5). **e** MDA-MB-231 cells were treated with nalbuphine and/or SC79 for 48 h and then subjected to wound healing analysis. Representative images (*left*) and statistical analysis (*right*) are shown (n = 3). Data are presented as mean ± SEM. *p-*value was determined by Student’s *t*-test and ANOVA (**c** and **d**) (*p < 0.05, **p < 0.01, ***p < 0.001). See also Additional file 6: Figure S5

The expression of AKT and NFκB was measured in nalbuphine-treated cells, and we found that nalbuphine decreased the production of p-AKT and p-NFκB in a dose-dependent manner by inhibiting phosphorylation, without changing the levels of AKT and NFκB (Fig. 5a). We then treated cells with SC79, an AKT agonist, to detect whether nalbuphine inhibited cancer stem-like traits and EMT through the AKT-NFκB signaling pathway. SC79 treatment promoted phosphorylation of AKT-NFκB and stem-like properties and reversed the nalbuphine-induced repression of AKT-NFκB phosphorylation and stem-like proteins under nalbuphine treatment (Fig. 5b). Another AKT agonist, IGF-1, also enhanced cancer stem-like traits and reversed the nalbuphine-decreased stem-like phenotype (Additional file 6: Figure S5A). SC79-treated cells exhibited increased sphere formation efficiencies in both diameter and numbers, even in the presence of nalbuphine (Fig. 5c). SC79 treatment of mice xenografted with a human mammary cancer cell line promoted tumor growth and reversed the nalbuphine-repressed tumorigenesis (Fig. 5d). Treatment with SC79 also reversed the increased expression of E-cadherin and prevented the decrease in N-cadherin, Vimentin and Snail caused by nalbuphine (Fig. 5b) and enhanced cell migration in the presence of nalbuphine (Fig. 5e). Previous study showed that PTEN inactivation resulted in AKT activation by phosphorylation [27] and treatment with SC79 resulted in more phosphorylated AKT and NFκB upon knockdown of PTEN (Additional file 6: Figure S5B). These findings suggested that nalbuphine inhibited breast cancer stem-like traits via the AKT-NFκB signaling pathway.

## Discussion

Pain is one of the most common and debilitating symptoms of cancer and markedly reduces a patient’s quality of life [28]. Adjuvant analgesics are given as part of the therapeutic strategy [29, 30], but some reports showed that opioid analgesics like morphine can contribute to tumor progression [5, 24, 31]. In this study, we found that the narcotic nalbuphine downregulated cancer stem-like properties and EMT of breast cancer cells by inhibiting the AKT-NFκB signaling pathway. Cancer stem-like properties and EMT have been proposed as the driving force for malignant transformation in various cancers, and are closely involved in drug resistance and poor prognosis [32, 33]. Lennon et al. showed that [d-Ala(2),N-Me-Phe(4),Gly(5)-ol]-enkephalin (DAMGO), morphine and fentanyl facilitated EMT in non-small cell lung cancer by activating the opioid receptors [34]. Our previous results also demonstrated that morphine and fentanyl induced breast cancer stem cell properties, and morphine treatment led to chemoresistance to doxorubicin and paclitaxel [2, 3]. Therefore, we looked for any analgesics that could inhibit cancer stem-like traits and EMT, and found nalbuphine, an inexpensive, non-controlled, opioid analgesic, that suppressed tumor-sphere formation of MDA-MB-231, MCF-7 and SK-BR-3 human breast cancer cell lines. Nalbuphine treatment downregulated the expression of stemness markers in both breast cancer cells and mice xenografted with human breast cancer cells. Nalbuphine also repressed the migration and invasion of breast cancer cells and repressed the EMT in vitro and in vivo by regulating the expression of the markers. Nalbuphine was capable of inhibiting the growth of several other types of tumor cells, with little or no effect on noncancerous breast cell lines. Taken together, our study demonstrates for the first time that nalbuphine inhibits cancer stem cell properties and EMT of breast cancer cells, and may suppress tumor progression in the treatment of breast cancer. Further investigation is needed.

The aberrant activation of the AKT-NFκB signaling pathway is associated with a variety of pathological alterations. Both AKT and NFκB play important roles in many cellular processes, including cell proliferation, apoptosis, migration, invasion, tumor angiogenesis and lipid metabolism [35–38]. Some studies have demonstrated that the AKT-NFκB signaling pathway is involved in the promotion of cancer stem-like traits, and is closely correlated with EMT [39–42]. Our results show that nalbuphine repressed AKT and NFκB activation, while the AKT-NFκB signaling agonist SC79 reversed the effects of nalbuphine. These data suggest that nalbuphine suppressed breast cancer stem cell properties and EMT through its effects on the AKT-NFκB signaling pathway; but, the exact mechanism(s) by which nalbuphine decreased cancer stem-like properties and EMT remains to be determined.

## Conclusions

Our findings illustrate a new role for nalbuphine in inhibiting cancer stem-like properties and EMT as well as relieving pain, which suggest the use of nalbuphine as an effective adjunct in breast cancer treatment.

## Additional files

Additional file 1: Supplemental methods. (DOCX 688 kb)

Additional file 2: **Figure S1.** Nalbuphine inhibits tumor cell proliferation. (**A**) SK-BR-3 cells were incubated with the indicated concentration of nalbuphine (Nal) for the indicated times and cell viability was measured using the MTT method (*n* = 3). (**B-C**) Colony formation of MCF-7 (**B**) and SK-BR-3 (**C**) cells treated with the indicated concentrations of Nal (n = 3). Data represent mean ± SEM. *p*-value was determined by Student’s *t*-test (*p < 0.05, **p < 0.01, ***p < 0.001). (DOCX 688 kb)

Additional file 3: **Figure S2.** Nalbuphine suppresses breast cancer stem-like traits. (**A**) SK-BR-3 cells were treated with nalbuphine for 48 h and levels of the indicated mRNAs were determined by RT-PCR (*n* = 3). (**B**) MDA-MB-231 and MCF-7 cells were treated with the indicated concentration of nalbuphine for 48 h and levels of the indicated proteins were determined by western blot (*n* = 3). (**C)** SK-BR-3 cells were treated with nalbuphine for 48 h and levels of the indicated proteins were determined by western blot (n = 3). (**D**) MDA-MB-231 cells were treated with nalbuphine for the indicated times, and levels of the indicated proteins were determined by western blot (n = 3). (**E**) Representative spheroid images derived from the Ctrl and Nal MCF-7 and SK-BR-3 cells (*upper*) (*n* = 3); Scale bar, 50 μm. Mammosphere sizes and number of mammospheres (*bottom*) (d > 50 μm) are shown. Data represent mean ± SEM. *p*-value was determined by Student’s *t*-test and ANOVA (E) (*p < 0.05, **p < 0.01, ***p < 0.001). (DOCX 563 kb)

Additional file 4: **Figure S3.** Morphine and fentanyl promote tumorigenesis. (**A**) MDA-MB-231 and MCF-7 cells were treated with nalbuphine (Nal), morphine (Mor), or fentanyl (Fen) for 48 h and levels of the indicated proteins were determined by western blot (*n* = 3). (**B**) Representative tumor image (*left*) from Ctrl, Nal, Mor and Fen mice; growth of Ctrl, Nal, Mor and Fen MDA-MB-231 tumors in mice (n = 3). Data represent mean ± SEM. *p*-value was determined by ANOVA (B) (*p < 0.05, **p < 0.01, ***p < 0.001). (DOCX 266 kb)

Additional file 5: **Figure S4.** Nalbuphine inhibits EMT and metastasis. (**A-B**) SK-BR-3 cells were treated with nalbuphine for 48 h and the levels of the indicated mRNAs and proteins were determined by RT-PCR (**A**) and western blot (**B**) (n = 3). (**C**) MCF-7 and SK-BR-3 cells were treated with nalbuphine for 48 h, and then subjected to wound healing analysis; representative images (*left*) and statistical analysis (*right*) are shown (n = 3). (**D**) MCF-7 and SK-BR-3 cells were treated with nalbuphine for 48 h and migration and invasion ability were determined by transwell assays; representative images (*left*) and statistical analysis (*middle and right*) are shown (n = 3). Data represent mean ± SEM. *p*-value was determined by Student’s *t-*test (*p < 0.05, **p < 0.01, ***p < 0.001). (DOCX 842 kb)

Additional file 6: **Figure S5.** Nalbuphine inhibits breast cancer stem-like properties through the AKT-NFκB pathway. (**A**) MDA-MB-231 cells were treated with nalbuphine and/or IGF-1 for 48 h and levels of indicated proteins were determined by western blot (n = 3). (**B**) MDA-MB-231 cells transfected with shPTEN were treated with SC79 for 48 h and levels of indicated proteins were determined by western blot (n = 3). (DOCX 445 kb)

Additional file 7: **Table S7.** IC50 of nalbuphine in MDA-MB-231, MCF-7 and SK-BR-3 cells. (XLSX 9 kb)

## Abbreviations

ATCC: American type culture collection
BPE: Bovine pit extract
CSCs: Cancer stem cells
DEAB: Diethylaminobenzaldehyde
DMEM: Dulbecco’s modified eagle medium
EMEM: Eagle’s minimum essential medium
EMT: Epithelial-mesenchymal transition
FACS: Fluorescence activated cell sorting
IC50: 50% inhibitory concentration
IHC: Immunohistochemical
MEBM: Mammary epithelium basal medium
MSC: Mesenchymal stem cell
NFkB: Nuclear factor kappa B
