# Supplementary material for: Nalbuphine suppresses breast cancer stem-like properties and epithelial-mesenchymal transition via the AKT-NFκB signaling pathway
Source: J Exp Clin Cancer Res. 2019 May 15;38:197. doi: 10.1186/s13046-019-1184-1 (PMC6521451; doi:10.1186/s13046-019-1184-1)
Supplement: Supplementary file 1 — Supplemental methods. (DOCX 688 kb) [file 13046_2019_1184_MOESM1_ESM.docx]

**Supplemental Methods**

**Cell proliferation assay**

The MTT (Sigma) assay was used to assess the growth of cells according to manufacturer’s directions. Cells (2-3 × 10^3^) were plated in 96-well flat-bottom plates in a final volume of 100 μl. After attachment, the cells were exposed to drugs for 24 to 72 hr and cell survival was determined spectrophotometrically at 490 nm.

**Colony formation**

Log-phase cultures were plated into 60 mm dishes (1×10^3^ cells per dish) and cultured at 37°C with 5% CO_2_. Cells were fed with fresh growth medium every 3 days. After 14 days of incubation, colonies were fixed with 4% paraformaldehyde (PFA), stained with crystal violet, and counted using Image J software. Each experiment was repeated three times.

**Wound healing**

Tumor cells were grown to confluence, and monolayers were then scratched in a straight line using a sterile pipet tip to mimic an incision wound. Cells were washed with PBS to remove debris and fresh culture medium was added, with or without 100 μM nalbuphine and incubated at 37°C. Wound closure was monitored by collecting digitized images at 24 hr after the scratch was performed, and the area of the scratch was determined using Image-J software. Data represent the extent of wound closure. All measurements were performed in triplicate.

**Transwell migration and invasion assays**

For measuring migration capability, 5 × 10^4^ cells pretreated with 100 μM nalbuphine for 48 hr were resuspended in culture medium and pipetted into the upper chamber of an 8 μm transwell insert in a 24-well plate (Corning Costar). DMEM supplemented with 10% FBS was added to the lower chamber as an ‘attractant’. After 24 hr incubation, the cells that had migrated through the membrane were fixed with 4% paraformaldehyle (Santa Cruz) and stained with DAPI (Shanghai Sangon Company). The stained cells were examined under a fluorescence microscope (Olympus) at 10× magnification and five random fields were counted. Results were the average of triplicate samples from three independent experiments.

For invasion assays, 8 × 10^4^ cells were placed into the upper transwell insert after coating the membrane with 50 μl of Matrigel (BD Biosciences) and incubated for 36 hours. Following steps were similar to migration assays.

**RT-PCR and Real-time RT-PCR**

Total RNA was extracted using TRIzol reagent (Invitrogen) and used to generate cDNA by EasyScript One-Step gDNA Removal and cDNA Synthesis SuperMix (Transgen) with an oligo-dT primer. RT-PCR was performed using Premix Taq (Takara) as recommended by the manufacturer. Real-time RT-PCR was performed using SYBR Select Master Mix (Life Technology) as recommended by the manufacturer. β-actin was used as the internal control. Primer sequence are listed below.

| **RT-PCR** | **sense (5′-3′)** | **antisense (5′-3′)** |
| --- | --- | --- |
| MYC | TCAAGAGGCGAACACACAAC | GGCCTTTTCATTGTTTTCCA |
| NANOG | CCTGTGATTTGTGGGCCTGA | CTCTGCAGAAGTGGGTTGTTTG |
| OCT4 | GAGAACCGAGTGAGAGGCAACC | CATAGTCGCTGCTTGATCGCTTG |
| SOX2 | GTGAGCGCCCTGCAGTACAA | GCGAGTAGGACATGCTGTAGGTG |
| E-cadherin | CAACGACCCAACCCAAGAA | CCGAAGAAACAGCAAGAGCA |
| N-cadherin | AAAGAACGCCAGGCCAAAC | GGCATCAGGCTCCACAGTGT |
| Vimentin | CGTCTCTGGCACGTCTTGAC | GCTTGGAAACATCCACATCGA |
| SNAIL | CGGAAGCCTAACTACAGCGA | GGACAGAGTCCCAGATGAGC |
| ACTB | TTGCCGACAGGATGCAGAAGGA | AGGTGGACAGCGAGGCCAGGAT |

**Western blotting**

Cells were lysed on ice in RIPA buffer and protein concentrations were determined using Coomassie brilliant blue. Equal amounts of protein were subjected to electrophoresis on 10% gradient SDS-PAGE gels and then transferred to nitrocellulose membranes (Millipore) for western blotting. The following primary antibodies were used: GAPDH (1:5,000 dilution, Proteintech, Cat#60004-1-Ig), SOX2 (1:1000 dilution, CST, Cat#14962), NANOG (1:1000 dilution, Abcam, Cat#ab80892), E-cadherin (1:1000 dilution, Proteintech, Cat#20874-1-AP), OCT4 (1:1000 dilution, CST, Cat#75463), Snail (1:1000 dilution, Abcam, Cat#ab180714), Vimentin (1:1000 dilution, Proteintech, Cat#10366-1-AP), N-cadherin (1:1000 dilution, Proteintech, Cat#22018-1-AP), MYC (1:1000 dilution, Abcam, Cat#ab32), AKT (1:1000 dilution, CST, Cat#4691), p-AKT (1:1000 dilution , CST, Cat#4060), NFκB (1:1000 dilution, CST, Cat#3034) and p-NFκB (1:1000 dilution, CST, Cat#3033). After primary antibody binding, blots were washed and horseradish peroxidase-conjugated goat anti-mouse or goat anti-rabbit IgG was used as secondary antibody (1:5,000 dilution, Thermo Fisher Scientific, Cat#31430 or Cat#31460). Antibody binding was detected with a WesternBright^TM^ECL kit (Advansta).

**Immunohistochemical staining and statistical analysis**

Paraffin-embedded tissue blocks were used for immunohistochemical staining. Paraffin-embedded tissue specimens were sectioned, deparaffinized in xylene and rehydrated. Antigen retrieval was performed using sodium citrate, and the sections were then processed using SPlink Detection Kits (OriGene) according to the manufacturer’s instructions. The sections were incubated with anti-SOX2 antibody (1:200 dilution), anti-OCT4 antibody (1:200 dilution), anti-NANOG antibody (1:200 dilution), anti-MYC antibody (1:200 dilution), anti-E-cadherin antibody (1:200 dilution), anti-N-cadherin antibody (1:200 dilution), anti-Vimentin antibody (1:200 dilution) or anti-Snail antibody (1:200 dilution) overnight at 4°C. Specimens were stained using a DAB kit (ZSGB-BIO) until the desired stain intensity was developed. Sections were then counterstained with hematoxylin, dehydrated, and mounted. Staining intensity and extent of SOX2, OCT4, NANOG, MYC, E-cadherin, N-cadherin, Vimentin, Snail expression were graded as follows: negative (0), bordering (1), weak (2), moderate (3), and strong (4). Extent of staining was also grouped into quantiles according to the percentage of high-staining cells fields: negative (0), 25% (1), 26-50% (2), 51-75% (3), and 76-100% (4). All immunohistochemical staining was evaluated and scored by at least two independent pathologists.

**Detection of alanine aminotransferase, aspartate aminotransferase, urea and creatinine**

Blood was collected from the medial canthal vein of mice and allowed to clot at room temperature for one hour. Samples were then centrifuged at 1000 rpm for 10 minutes at 4°C, and the supernatant sera collected for examination. Detection of analytes was performed using an alanine aminotransferase assay kit (C009-1, Njjcbio), aspartate aminotransferase assay kit (C010-1, Njjcbio), urea assay kit (C013-2, Njjcbio) and creatinine assay kit (C011-2, Njjcbio) according to the manufacturer’s protocol.

**Gene knockdown with short-hairpin RNA (shRNA)**

Knockdown of genes was performed with specific shRNAs delivered using a lentiviral system (Sigma-Aldrich Corp.) according to the instructions provided by the manufacturer. In brief, to generate the lentivirus containing the specific shRNA, shPTEN-1-F: CCGGCCAGCTAAAGGTGAAGATATACTCGAGTATATCTTCACC TTTAGCTGGTTTTT, shPTEN-1-R: AATTAAAAACCAGCTAAAGGTGAAGATA TACTCGAGTATATCTTCACCTTTAGCTGG, shPTEN-2-F: CCGGCAGTATAGAG CGTGCAGATAACTCGAGTTATCTGCACGCTCTATACTGTTTTT, shPTEN-2-R: AATTAAAAACAGTATAGAGCGTGCAGATAACTCGAGTTATCTGCACGCTCTATACTG. 293T cells were co-transfected with 2.5 mg pMD2.G and 7.5 mg psPAX2-compatible packaging plasmids and 10 mg of pLKO.1 plasmid bearing the specific shRNA for 24 hours. Culture medium containing the generated lentiviruses was collected and stored at -80℃ as aliquots for further use. To deliver the specific shRNA construct, approximately 30% confluent cells were infected with lentiviruses bearing the specific shRNA in growth medium containing 8 mg/ml polybrene and were incubated at 37℃ for 48 hours. Transfected cells were subsequently selected with 2 mg/ml puromycin.
