# Supplementary material for: Nalbuphine suppresses breast cancer stem-like properties and epithelial-mesenchymal transition via the AKT-NFκB signaling pathway
Source: J Exp Clin Cancer Res. 2019 May 15;38:197. doi: 10.1186/s13046-019-1184-1 (PMC6521451; doi:10.1186/s13046-019-1184-1)
Supplement: Supplementary file 2 — Figure S1. Nalbuphine inhibits tumor cell proliferation. (A) SK-BR-3 cells were incubated with the indicated concentration of nalbuphine (Nal) for the indicated times and cell viability was measured using the MTT method (n = 3). (B-C) Colony formation of MCF-7 (B) and SK-BR-3 (C) cells treated with the indicated concentrations of Nal (n = 3). Data represent mean ± SEM. p-value was determined by Student’s t-test (*p < 0.05, **p < 0.01, ***p < 0.001). (DOCX 688 kb) [file 13046_2019_1184_MOESM2_ESM.docx]

**Figure S1. Nalbuphine inhibits tumor cell proliferation.**
