# Supplementary material for: Nalbuphine suppresses breast cancer stem-like properties and epithelial-mesenchymal transition via the AKT-NFκB signaling pathway
Source: J Exp Clin Cancer Res. 2019 May 15;38:197. doi: 10.1186/s13046-019-1184-1 (PMC6521451; doi:10.1186/s13046-019-1184-1)
Supplement: Supplementary file 3 — Figure S2. Nalbuphine suppresses breast cancer stem-like traits. (A) SK-BR-3 cells were treated with nalbuphine for 48 h and levels of the indicated mRNAs were determined by RT-PCR (n = 3). (B) MDA-MB-231 and MCF-7 cells were treated with the indicated concentration of nalbuphine for 48 h and levels of the indicated proteins were determined by western blot (n = 3). (C) SK-BR-3 cells were treated with nalbuphine for 48 h and levels of the indicated proteins were determined by western blot (n = 3). (D) MDA-MB-231 cells were treated with nalbuphine for the indicated times, and levels of the indicated proteins were determined by western blot (n = 3). (E) Representative spheroid images derived from the Ctrl and Nal MCF-7 and SK-BR-3 cells (upper) (n = 3); Scale bar, 50 μm. Mammosphere sizes and number of mammospheres (bottom) (d > 50 μm) are shown. Data represent mean ± SEM. p-value was determined by Student’s t-test and ANOVA (E) (*p < 0.05, **p < 0.01, ***p < 0.001). (DOCX 563 kb) [file 13046_2019_1184_MOESM3_ESM.docx]

**Figure S2. Nalbuphine suppresses breast cancer stem-like traits.**
