# Supplementary material for: Nalbuphine suppresses breast cancer stem-like properties and epithelial-mesenchymal transition via the AKT-NFκB signaling pathway
Source: J Exp Clin Cancer Res. 2019 May 15;38:197. doi: 10.1186/s13046-019-1184-1 (PMC6521451; doi:10.1186/s13046-019-1184-1)
Supplement: Supplementary file 4 — Figure S3. Morphine and fentanyl promote tumorigenesis. (A) MDA-MB-231 and MCF-7 cells were treated with nalbuphine (Nal), morphine (Mor), or fentanyl (Fen) for 48 h and levels of the indicated proteins were determined by western blot (n = 3). (B) Representative tumor image (left) from Ctrl, Nal, Mor and Fen mice; growth of Ctrl, Nal, Mor and Fen MDA-MB-231 tumors in mice (n = 3). Data represent mean ± SEM. p-value was determined by ANOVA (B) (*p < 0.05, **p < 0.01, ***p < 0.001). (DOCX 266 kb) [file 13046_2019_1184_MOESM4_ESM.docx]

**Figure S3. Morphine and fentanyl promote tumorigenesis.**
