# Supplementary material for: Nalbuphine suppresses breast cancer stem-like properties and epithelial-mesenchymal transition via the AKT-NFκB signaling pathway
Source: J Exp Clin Cancer Res. 2019 May 15;38:197. doi: 10.1186/s13046-019-1184-1 (PMC6521451; doi:10.1186/s13046-019-1184-1)
Supplement: Supplementary file 5 — Figure S4. Nalbuphine inhibits EMT and metastasis. (A-B) SK-BR-3 cells were treated with nalbuphine for 48 h and the levels of the indicated mRNAs and proteins were determined by RT-PCR (A) and western blot (B) (n = 3). (C) MCF-7 and SK-BR-3 cells were treated with nalbuphine for 48 h, and then subjected to wound healing analysis; representative images (left) and statistical analysis (right) are shown (n = 3). (D) MCF-7 and SK-BR-3 cells were treated with nalbuphine for 48 h and migration and invasion ability were determined by transwell assays; representative images (left) and statistical analysis (middle and right) are shown (n = 3). Data represent mean ± SEM. p-value was determined by Student’s t-test (*p < 0.05, **p < 0.01, ***p < 0.001). (DOCX 842 kb) [file 13046_2019_1184_MOESM5_ESM.docx]

**Figure S4. Nalbuphine inhibits EMT and metastasis.**
