# Supplementary material for: Nalbuphine suppresses breast cancer stem-like properties and epithelial-mesenchymal transition via the AKT-NFκB signaling pathway
Source: J Exp Clin Cancer Res. 2019 May 15;38:197. doi: 10.1186/s13046-019-1184-1 (PMC6521451; doi:10.1186/s13046-019-1184-1)
Supplement: Supplementary file 6 — Figure S5. Nalbuphine inhibits breast cancer stem-like properties through the AKT-NFκB pathway. (A) MDA-MB-231 cells were treated with nalbuphine and/or IGF-1 for 48 h and levels of indicated proteins were determined by western blot (n = 3). (B) MDA-MB-231 cells transfected with shPTEN were treated with SC79 for 48 h and levels of indicated proteins were determined by western blot (n = 3). (DOCX 445 kb) [file 13046_2019_1184_MOESM6_ESM.docx]

**Figure S5. Nalbuphine inhibits breast cancer stem-like properties through the AKT-NFκB pathway.**
